# Adolescents with Type 1 Diabetes: parental perceptions of child health and family functioning and their relationship to adolescent metabolic control

**DOI:** 10.1186/1477-7525-11-50

**Published:** 2013-03-22

**Authors:** Susan M Moore, Naomi J Hackworth, Victoria E Hamilton, Elisabeth P Northam, Fergus J Cameron

**Affiliations:** 1 Faculty of Life & Social Sciences, Swinburne University of Technology, PO Box 218, Hawthorn, Vic, 3122, Australia; 2 Parenting Research Centre, Level 5, 232 Victoria Parade, East Melbourne, Vic, 3002, Australia; 3 Murdoch Children’s Research Institute, Flemington Rd, Parkville, Vic, 3052, Australia; 4 Department of Psychology, Royal Children’s Hospital, Flemington Rd, Parkville, Vic, 3052, Australia; 5 Melbourne School of Psychological Sciences, Redmond Barry Building, The University of Melbourne, Melbourne, Vic, 3010, Australia; 6 Department of Endocrinology and Diabetes, Royal Children's Hospital, Flemington Rd, Parkville, Vic, 3052, Australia

**Keywords:** Type 1 diabetes, Adolescent metabolic control, Family dynamics

## Abstract

**Background:**

Adolescents with Type 1 diabetes (T1D) show less effective metabolic control than other age groups, partly because of biological changes beyond their control and partly because in this period of developmental transition, psychosocial factors can militate against young people upholding their lifestyle and medical regimens. Parents have an important role to play in supporting adolescents to self-manage their disease, but resultant family tensions can be high. In this study, we aimed to assess family functioning and adolescent behaviour/ adjustment and examine the relationships between these parent-reported variables and adolescent metabolic control (HbA1c), self-reported health and diabetes self-care.

**Method:**

A sample of 76 parents of Australian adolescents with T1D completed the Child Health Questionnaire –Parent form. Their adolescent child with T1D provided their HbA1c level from their most recent clinic visit, their self-reported general health, and completed a measure of diabetes self-care.

**Results:**

Parent-reported family conflict was high, as was disease impact on family dynamics and parental stress. Higher HbA1c (poorer metabolic control) and less adequate adolescent self-care were associated with lower levels of family functioning, more adolescent behavioural difficulties and poorer adolescent mental health.

**Conclusions:**

The implication of these findings was discussed in relation to needs for information and support among Australian families with an adolescent with T1D, acknowledging the important dimension of family functioning and relationships in adolescent chronic disease management.

## Background

Type 1 diabetes (T1D) is a serious, life-long autoimmune condition where an individual’s immune system attacks the beta cells in the pancreas responsible for producing insulin, the hormone required to convert food into energy [1]. Management of T1D requires a strict daily regimen of insulin injections, finger-prick blood tests and dietary monitoring. People with Type 1 diabetes must be constantly vigilant for episodes of hyperglycemia (extremely high blood sugars) or hypoglycemia (extremely low blood sugars), both of which can be life threatening. T1D is associated with serious long-term health complications including heart disease, vascular disease, kidney disease, blindness (retinopathy), and neuropathy [2]. A number of large scale trials, for example, the Diabetes Control and Complications Trial, DCCT Research Group [3], have demonstrated that with intensive management these complications can be minimized, however optimal metabolic control is elusive. While much research has focused on advancements in the clinical management of diabetes, particularly technological advancements in diabetes care, psychosocial, behavioural and contextual factors are less often documented in clinical care [4]. Therefore further investigation into the influence of family dynamics and family context on adolescent diabetes self-management is warranted.

Disease onset for TID is rapid and usually occurs before or during adolescence [1]. This is a time when young people are also negotiating major physical, cognitive and psychological changes, and are at heightened vulnerability for engaging in risk taking behaviours (e.g., alcohol and other drug use) and for developing mental health problems [5,6]. While the rate of risk taking behavior has been found to be similar for adolescents with and without T1D, a general propensity toward risky behaviour in adolescents with T1D has been linked to poorer self care, and in turn to poor mental health and metabolic outcomes [6,7].

Evidence suggests that adherence to diabetes self care regimes during adolescence is particularly poor [8]. The pressures and changes of normal adolescent development can conflict with the self- awareness, restraint and orderliness needed to manage living with a chronic disease, and these tensions create a platform for significant personal and family stress and even mental illness [9].

What is the appropriate role of parents in helping their child with T1D manage the transition to autonomous self-care? Parenting an adolescent can be challenging enough, but parents of children with a chronic disease have the added worry that their child will neglect self-care routines in their desire to do what other teenagers do – appear ‘cool’, experiment with alcohol, stay out late, compete with peers on the sporting field, lose weight – all those activities that can jeopardize dietary, life style and glucose/insulin self-management. Perhaps unsurprisingly, parents have been shown to experience anxieties and stress in relation to their child’s illness within four weeks of a Type 1 diabetes diagnosis in the family [10]. Dashiff et al. [11] in a qualitative study of 40 parents of adolescents with T1D, found parents indicated that they experienced ongoing struggle, worry and frustration about their parenting role. They reported providing support for their child’s transition to self-management mainly via the strategies of rewarding positive behaviours, providing reminders and granting more freedom. They also reported being aware that child self-management was poorer when they as parents engaged in scolding, judging, checking, nagging, or becoming emotional, but many parents found it difficult to stop these behaviours because of their worry about non-adherence. Similarly, in a study of parents of younger children with T1D, Wilson, DeCourcey and Freeman [12] found children’s non-adherence, particularly in relation to mealtime misbehaviour, was associated with parents’ self reported over-reactive discipline.

An emotionally supportive and accepting parenting style (parental responsiveness) is well-documented as having enduring implications in improved quality of life for children and adolescents with T1D [13]. In a sample of 81 adolescents with T1D and their parents, Botello-Harbaum and colleagues [13] found that over a 12-month period, parental responsiveness continued to promote greater self-reported quality of life in their child. On the other hand, Drew et al. [14] showed depleted family resources – emotional as well as financial – were associated with poorer metabolic control among 252 adolescents with T1D. Maintaining the daily routines necessary for diabetes management was less successful in families with lower parental acceptance (communication of love, warmth, acceptance) and higher levels of parental depressive symptoms, especially when there was the added stress of low family income. Rosenberg and Shields’ [15] finding that stronger parental attachment was related to better metabolic control among 31 adolescents with T1D provides additional evidence for the importance of positive family dynamics as a background for young people learning self-management of chronic disease. That maintaining such positive dynamics is not an easy task is reflected in an Indian study of 50 parents of adolescents with T1D [16]. Scores on the General Health Questionnaire (GHQ) showed 17 out of the 50 parents had a diagnosable psychiatric disorder, and nearly two-thirds showed tendencies toward psychological dysfunction. While the authors report that these effects are likely to be weaker in cultures where diabetes is less stigmatized, nevertheless they point to the stressful nature of having a chronically ill adolescent in the family, not just for the child but also for parent. Such findings highlight the apparent cyclical nature of successful chronic disease management of a family member, and mental and emotional wellbeing within the family as a whole.

Indeed, pediatric T1D is often characterized as a ‘family disease’ because of the important role of family interactions and parental support [17]. In a study of 187 adolescents with T1D and their parents, Williams et al. [17], in a US study, found close links between poor adolescent glycaemic control, family conflict and parental psychological distress. They suggest that effects are interactive, such that poorer family functioning is associated with poorer adolescent self-management, which in turn leads to more family conflict and distress.

In a Dutch study by deWit et al. [18], parents rated the health and functioning of their adolescent with T1D using the Child Health Questionnaire (CHQ). Adolescents rated by their parents as healthier functioning and better behaved were more likely to have better glycaemic control as assessed by their HbA1c level. In particular, better metabolic control was correlated with parent-rated physical and mental health summary scores, child behaviour, the impact of their child’s disease on parents (both emotionally and time-wise) and participation in family activities. In more than 80 percent of their sample of 91 adolescents, metabolic control was sub-optimal, that is, HbA1c levels were above 7.5 percent, increasing the possibilities of these young people developing disease complications [18]. That lower psychosocial well-being, more externalising behaviour, and greater family and parental impact were associated with weaker metabolic control again points to probable interactive relationships between disease control and family functioning, as suggested by Williams et al. [17].

The aim of the current study was to attempt replication of studies showing the interactive effects of adolescent disease control and family functioning among a sample of Australian adolescents with T1D and their parents. The incidence of paediatric TID in Australia (and New Zealand) is particularly high, especially in comparison with other Western Pacific nations [19]. Nearly three out of every thousand Australian children aged 10 to 14 (256 per 100,000) have been diagnosed with this disease, a prevalence that is rising year by year [1]. Given that diabetes has been described as ‘one of the leading threats to the health of Australians’ (p.1) [20], it is important to examine modifiable social and psychological factors relating to the disease within their specific contexts. While there is increasing emphasis being placed on the influence of T1D on family dynamics and parent psychosocial health, very few Australian studies have directly explored this relationship, or used both parental and adolescent reports of health and well-being. Additionally, studies from other countries examining these issues often use relatively small sample sizes, increasing the importance of replication particularly across a range of contexts.

Specifically, we aimed to assess family functioning, parent self-reported emotional wellbeing, and parental evaluation of child behaviour and adjustment via the Child Health Questionnaire [21], and examine the relationships between these parent-reported variables and metabolic control (HbA1c), child-reported health and self-care. Our predictions, based on previous literature from other developed nations, were that higher HbA1c levels and poorer adolescent self-care would be correlated with poorer parent-reported child behaviour, poorer child mental health and lower levels of family functioning and parent emotional wellbeing.

## Method

### Participants

Seventy-six parents of adolescents with T1D, and the adolescents themselves, were recruited through the Royal Children’s Hospital (Melbourne, Australia) diabetes clinics (59 mothers, 17 fathers; 44 adolescent girls, 32 adolescent boys; mean age of adolescents =14.57 years, range 12–18 years, SD = 1.67 years). The mean duration of diabetes for the full sample was 5.84 years (SD = 3.79 years). Sixteen adolescents (21%) reported receiving insulin via 2 injections per day, 37 (49%) via 3 or more injections per day and 22 (29%) via a continuous insulin delivery system (pump). One person did not report their treatment regime.

### Measures

#### Parent questionnaire

The parent questionnaire assessed a range of demographics (e.g., age, post-code, education) and parent perceptions of their child’s health and wellbeing using the Child Health Questionnaire Parent report (CHQ-PF50) [22]. The CHQ-PF50 comprises 15 subscales: global health (GGH), a one-item overall rating; physical functioning (PF), a scale mainly concerned with energy levels; role/social limitations-emotional/behavioural (REB), assessing limitations to the child’s school or peer group activities due to emotional or behavioural issues; role/social-limitations –physical (RP), assessing limitations to school and peer group activities due to physical problems; bodily pain/discomfort (BP); behaviour (BE), generally assessing negative behaviours such as arguing, anti-social activities, temper; general behaviour (GBE), a one-item overall rating of behaviour; mental health (MH), a scale assessing the child’s degree of sadness, anxiety, etc.; self esteem (SE); general health perceptions (GH), a measure of the level of illness experienced by the child; change in health (CH), a one–item rating; parent impact-emotional (PE), the extent to which the parent worries about and is emotionally affected by the child’s condition; parent impact-time (PT), the extent to which parents believe they must give up time to attend to the child’s condition; family activities (FA), a scale assessing how much parents believe their family is impacted in terms of activities and atmosphere by the child’s condition; and family cohesion (FC), a one-item global rating. The parent is asked to recall the preceding 4-week period for all subscales except GGH, GH, CH, and FC subscales. The recall stem for CH is ‘compared to last year’. The others are ‘in general’ with no specific recall time used. Scores on each subscale range from 0–100 with higher scores reflecting better health. Parents respond to items on 4-, 5- or 6-point scales with descriptions of the rating points varying across subscales, for example ‘very satisfied’ to ‘very unsatisfied’, or ‘all of the time’ to ‘none of the time’. The CHQ-PF50 has been shown to have high internal consistency and validity in both clinical and general population settings in Australia and the US [21-23]. For the current sample, alpha reliability co-efficients for subscales (with more than one item) were good to adequate, as follows: PF (0.92); RP (0.90); BP (0.92); GH (0.64); REB (0.95); BE (0.84); MH (0.87); SE (0.84): PE (0.84); FA (0.90).

#### Adolescent questionnaire

A self report questionnaire was used to assess a range of demographics (e.g., age, education, living arrangements) and health-related factors including a self-rated one-item measure of general health, with ratings ranging from poor = 1 to excellent =5. Additionally, diabetes self care was assessed using the Diabetes Self Care Inventory (SCI-R) [24], a 15-item scale. Responses are rated on a 5-point Likert scale ranging from 1 = Never to 5 = Often. The scale has demonstrated strong internal consistency of *α* = .80 or higher [24-26], and achieved adequate internal consistency in the present sample (*α* = .77). Scores were summed with higher scores indicating a higher level of diabetes-relevant self-care.

Glycated haemoglobin level (HbA1c), as recorded during the clinic visit in which the study was carried out, was used as an indicator of metabolic control. HbA1c, measured by blood test, is a measure of glycemic control based on average blood glucose concentration levels in the 3–4 month period prior to the HbA1c test [27,28].

### Procedure

Participants were recruited through the Melbourne Royal Children’s Hospital rural and urban Outpatients Clinics. Recruitment occurred over a six-month period subsequent to ethical approval from the Royal Children’s Hospital Human Research Ethics Committee and the Swinburne University of Technology Human Research Ethics Committee. Participants (adolescents and parents) completed questionnaires while attending their usual Diabetes Outreach Clinic visit or while attending their hospital clinic visit prior to attending a residential camp program for youths with T1D. Although the response rate of adolescents or families approached was not recorded, there were 76 completed parent questionnaires received from the 91 families in which an adolescent had submitted a completed questionnaire, a response rate of 84% for parents.

During the course of questionnaire administration, adolescent participants were asked to copy their current HbA1c result (done on arrival at the diabetes clinic) from their patient record card directly onto the questionnaire. This mode of reporting of HbA1c was selected due to difficulties in obtaining ethics approval to directly access participant medical records. As the HbA1c result was transferred directly from the record card onto the questionnaire immediately after the HbA1c test, it was considered to be a reliable measure of HbA1c. In 57 cases, *both* parent and child reported the child’s HbA1c on their questionnaires; the correlation between reports was 0.97, suggesting high reliability of recording.

A plain language statement of research was provided and consent obtained from both the young person and their parent to be part of the study. Completed questionnaires were returned to the researcher in sealed envelopes.

### Analyses

Mean CHQ-PF scores of the sample were compared with mean scores for normative (non-clinical) samples of parents of 13–15 year olds and 16–18 year olds, using t-tests to assess significance of differences. Correlations between HbA1c, child-rated general health and self-care and parent-rated child behaviour (CHQ subscales), were calculated to test predictions that higher HbA1c levels, poorer adolescent health and self-care would be correlated with poorer parent-reported child behaviour and mental health and lower levels of family functioning.

## Results and discussion

HbA1c values for the adolescents in the sample were sub-optimal: 74.6% had values >7.5 and 52.1% had values >8.0, a similar finding to deWit et al. [18] in their study of Dutch adolescents, and Stewart et al. [8] researching US young people with TID. The mean HbA1 value was 8.55 (SD = 1.53; range 5.8-14.0). Nevertheless these adolescents on average self-rated their general health as good to very good (mean 3.70, SD = 0.70 on a 5-pont scale with 5 = excellent; range 2–5). Within this sample, neither age nor gender was significantly related to HbA1c level.

Mean scores for parent-rated CHQ scales in comparison with manualised norms for parents of children with age ranges 13–15 years and 16–18 years [23] are shown in Table 1, along with t-test results indicating significance of the differences between the samples. Mean scores for the T1D group were lower (indicating poorer health/well-being) on 7 of the 12 variables in comparison with the 13–15 normative group, and lower on 6 of these variables for the 15–18 group. The largest differences were evident for general health perceptions (GH), emotional impact on parents (PE) and impact on family activities (FA). The TID adolescents were also rated significantly lower than the normative groups on emotional/ behavioural limitations, mental health, time impact on parents and self esteem (in comparison with the younger normative group only). These findings differ somewhat from those of deWit et al. [18] who also used the CDQ with parents of adolescents with TID. These researchers, like us, found significant differences between TID adolescents and healthy controls on general health, but unlike the current study did not find clinical-control group differences on family impact or adolescent mental health variables. DeWit et al. however note that their sample is atypical of previous studies showing higher depression rates in adolescents with Type 1 diabetes [29-31]. Their findings may also reflect between-country differences (for example in family resources or service provision), given that researchers from India [16] and the US (e.g., [10,17]) have found higher than normative rates of family stress (for example reflected by high rates of parental depression and anxiety) in families where an adolescent has TID.

**Table 1:** Current sample CHQ-PF mean scores compared with norms

**CHQ subscales**	**Current sample**		**Normative sample**^**1**^			**Normative sample**^**1**^		
	**Australian**		**N = 95**			**N = 63**		
	**T1D**	**N = 76**	**13-15 yrs**			**16-18 yrs**		
	**Mean**	**SD**	**Mean**	**SD**	**t**	**Mean**	**SD**	**t**
Age (in years)	14.6	1.67						
GGH (Global Health)	81.78	19.57						
PF (Physical Functioning)	94.07	15.70	95.4	15.2	0.56	93.4	16.8	0.24
RP (Physical Limitations)	89.69	20.72	93.0	21.7	1.02	94.2	18.5	1.34
BP (Bodily Pain/Discomfort)	78.00	24.71	81.5	17.7	1.04	76.5	25.0	0.35
GH (General Health Perceptions)	60.76	17.99	73.0	17.8	4.44^***^	72.8	21.3	3.61^**^
CH (Health change)	75.16	21.00						
REB (Emotional/Behav Limitations)	83.04	27.72	91.9	21.0	2.31^*^	91.5	19.8	2.03^*^
BE (Behaviour)	69.54	21.07	72.0	19.2	0.79	69.5	23.5	0.01
GBE (General Behaviour)	78.00	25.35						
MH (Mental Health)	72.18	18.97	78.9	15.8	2.48^*^	80.4	19.1	2.53^*^
SE (Self Esteem)	70.17	19.07	77.2	18.9	2.40^*^	75.1	22.2	1.41
PE (Parent Impact-Emotion)	59.10	30.56	77.9	21.1	4.56^***^	80.0	21.5	4.66^***^
PT (Parental Impact-Time)	76.74	29.29	86.0	21.9	2.29^*^	89.5	22.8	2.82^**^
FA (Family Activities)	68.56	25.72	90.3	19.2	6.13^***^	94.8	16.1	7.03^***^
FC (Family Cohesion)	69.54	23.86	73.5	22.1	1.11	71.9	23.5	0.58

^1^ HealthActCHQ (2008); ^*^p < .05; ^**^p < .01; ^***^p < .001.

Focusing on some specific items from the CHQ-PF that target family conflict, 11% of parents said their adolescent argued very often, 23% often and 39% sometimes, while 44% said their adolescent had tantrums or showed hot temper (7% very often; 8% often; 29% sometimes). Over half (53%) said their adolescent caused tension in the home, or was a source of arguments (54%). Dashiff et al.’s [11] analysis of parents’ difficulties in trying to stay positive while encouraging their adolescents with TID to adhere to self-care routines provides a picture of how some of these tensions and conflicts can manifest. Young people who are striving towards independence from parental restraints can resent reminders or strictures regarding their health, even when they understand the importance of these; parents on the other hand can allow anxiety about their child’s health to interfere with their more adaptive parenting strategies. Conflict and non-compliance may be the outcome, as in Wilson et al.’s [12] study where ‘over-reactive’ discipline on the part of parents was associated with non-adherence to self care regimes by their TID children.

Correlations of parents’ Child Health Questionnaire scores with adolescents’ metabolic control, self-ratings of general health and self-care are shown in Table 2.

**Table 2:** Correlations between CHQ-PF subscales and HbA1c, adolescent self-rated general health and self-care scores

**Scales**^**2**^	**HbA1C**	**GHC**	**SC**
**Child-Rated variables**			
GHC (General Health)	-.10	1	**.40**
SC (Self Care)	**-.38**	**.40**	1
**Parent-Rated Physical Variables**			
GGH (Global Health)	-.18	**.43**	**.24**
PF (Physical Functioning)	-.20	.20^1^	.12
RP (Physical Limitations)	-.19	.11	.17
BP (Bodily Pain/Discomfort)	**-.25**	.05	.08
GH (General Health Perceptions)	-.10	**.33**	.13
CH (Change in Health)	-.10	**.27**	.06
**Parent-Rated Behavioural/Emotional Variables**			
REB (Emotional/Behavioural Limitations)	**-.24**	.**26**	.22^1^
BE (Behaviour)	**-.36**	**.38**	**.37**
GBE (General Behaviour)	-.12	**.24**	**.35**
MH (Mental Health)	**-.36**	**.32**	**.41**
SE (Self Esteem)	**-.28**	**.30**	**.26**
**Parent-Rated Family Variables**			
PE (Parental Impact-Emotional)	-.23^1^	.26	.22^1^
PT (Parental Impact-Time)	**-.29**	.13	.19
FA (Family Activities)	**-.27**	**.29**	**.27**
FC (Family Cohesion)	-.01	**.33**	**.25**

^1^ p < .1; bolded correlations p < .05 or better; ^2^ all scales scored so high scores represent better health.

The adolescents’ ratings of their own degree of self-management of diabetes (self-care) were significantly and positively related to their metabolic control (lower HbA1c levels), and to how healthy they felt in general, supporting findings of several previous studies (e.g., [6,7]). Interestingly, their HbA1c levels did not correlate with their overall ratings of how healthy they felt, underscoring the difficulties of maintaining optimum glycaemic levels in the absence of early warning signals of unsafe levels.

Parental ratings of their adolescent’s physical health were also not strongly associated with the adolescent’s metabolic control. The CHQ subscales GGH, PF, RP, BP, GH and CH are parent assessments of different aspects of their child’s physical health and functioning. Apart from Bodily Pain/Discomfort (which is generally readily observable), these scales did not significantly correlate with metabolic control, that is, they did not relate to the adolescent’s diabetic health. Three of the six parent-rated measures were significantly correlated with the child’s rating of his or her general health (and one further correlation approaches significance), so that parents and children were in some (relatively weak) agreement as to the adolescent’s physical health and functioning, but for neither adolescents nor their parents were general health ratings related to HbA1c. As well, adolescents’ scores on the diabetes self-care measure were only weakly associated with parent measures of child physical health (one significant correlation only).

Parent-rated child behaviour (subscales REB, BE, GBE) was a much stronger correlate of metabolic control than the physical health subscales of the CHQ. Although the one-item global rating of child behaviour did not correlate with HbA1c, both of the scales assessing behavioural difficulties (emotional/behavioural limitations on school work or peer interaction, and general behavioural dysfunction) were significantly correlated with metabolic control, so that as predicted, more antisocial and difficult behaviour was linked with weaker glycaemic control, worse self-care and poorer adolescent-rated general health, a finding reflected in previous research (e.g., [6-9]). Similarly as predicted, both parent-rated mental health indicators (MH, SE) were significantly correlated with metabolic control, child-rated health and self-care. Poorer mental health and lower self-esteem linked to poorer child health outcomes and less effective self-care, again similar to findings from studies in other western nations [17,18].

Finally, parent ratings of the impact of the child’s illness on themselves and the family in general (PE, PT, FA, FC) also related as predicted to adolescent metabolic control, self-care and general health. When metabolic control and adolescent self-care were poorer, parents reported feeling more emotionally burdened, family activities were more likely to be curtailed, and family dynamics were less positive. This strong finding in our Australian study is in line with research from around the world. Adolescent adherence to self-care regimes and/or glycaemic control are better when parents use ‘responsive’ influencing strategies [13], have greater resources including income, warmth and better mental health [14,15], are more supportive [13] and less conflicted or distressed [17,18].

## Conclusions

Australian adolescents with T1D show less effective metabolic control than other age groups (e.g., [32]). Difficulties in maintaining acceptable HbA1c levels occur in part because of the rapid biological changes characteristic of this age group, which complicate glucose and insulin regulation [18]. Even when general health is optimized and diabetes self-care is conscientious, adolescents may still experience diabetes-related health events like hypoglycemia, or they may sustain higher HbA1c levels than are conducive to avoiding long-term complications of the disease. Perhaps because of these complications, Australian adolescents and parents in our sample did not on the whole relate their general health and physical functioning to their diabetes health.

There are, in fact, minimal immediate symptoms or signs that HbA1C levels are sub-optimal. The best adolescents can do is maintain their self-care routines as carefully as they can, a difficult task likely to be facilitated by parental support, acknowledgement and reward. Given the issues of time, privacy and trust, it is not always possible for parents to directly observe their adolescent engaging in their diabetes self-care activities. This has the potential to heighten parents’ anxiety about their child’s welfare and therefore increase family conflict [13,14]. In the current study, around half the parents reported arguments, temper displays, and strained relationships with their T1D adolescent son or daughter, with about 10% indicating these happened frequently. Additionally, parental and family impact scores on the CDQ-PF demonstrated much more distressed family functioning than for the normative samples [23], suggesting these conflicts and stresses are over and above those experienced by having a healthy adolescent in the home.

Parent judgments of family dynamics, child behaviour and mental health were better indicators of their adolescent’s metabolic control than assessments of their child’s physical health, as indicated by the correlations in Table 2. What does this mean? One possibility is that when parents perceive their adolescent is neglecting self-care routines, they become more anxious and less likely to adopt positive parenting strategies, which in turn can exacerbate adolescent oppositional, aggressive or defiant behaviour, leading to greater neglect of self-management with corresponding rises in HbA1c. In this model, non-conforming adolescent behaviour leads to less than effective parental strategies that in turn lead to worse behaviour (and health outcomes) on the part of the adolescent. Another possibility begins with parental anxiety and stress leading to overprotection and/or parental limitations on their adolescent’s moves toward independence. In this model, young people with T1D may be less competent to take on the responsibility for their own health because they have not been given the tools or opportunity to do so. Probably, as Williams et al. [17] imply, the correlations represent two-way causation, with adolescent non-adherence (or parent perception of it) exacerbating parental concern and family stress, which in turn has effects on adolescent mental health, self-esteem, behavioural compliance and family dynamics. These effects, through neglect of self-care, are associated with less than optimal metabolic control.

It is worth noting that families participating in the current study were recruited while attending their routine diabetes clinic visits and therefore by definition were engaged with their diabetes care. Furthermore parents attended with their adolescents, indicating their support. It is not clear the degree to which these findings will generalise to those families who are less engaged with medical care and each other, but it is certainly of interest that even in supportive families the stresses are high. Additionally, the sample of parents was relatively small; limiting the extent to which more sophisticated statistics (e.g., regression analyses or structural equation modelling) could be used. However, the findings do indicate that further investigation in a larger sample is warranted, particularly in relation to teasing out the processes through which family tensions and adolescence non-adherence to care regimes interact. It is also well to remember that difficulties with maintaining HbA1c levels are multi-causal, and some of these causes are beyond the control of adolescents or their parents. Monitoring is the key, a difficult task at any age but especially so in the adolescent transition years.

The implications of these data relate to needs for information and support for Australian families with an adolescent with T1D. This is particularly important given the degree of stress shown by the families in this study, for example in relation to the parents of Danish adolescents with TID [18]. Parents without the requisite information and on-going support can find themselves overwhelmed with fears and worries about their adolescent, which in turn can lead to angry confrontations with children who are perceived as jeopardizing their own health. Adolescents can feel they are being patronized or not trusted, especially when they are trying hard to maintain good self-care regimes but do not always successfully manage the difficult task of satisfactory metabolic control. As well, adolescents can feel the unfairness of having to curtail their activities in ways not understood by peers. These feelings can give rise to anger, anxiety or depression, further barriers to good family communication. The findings of the current study suggest the importance not only of providing information on diabetes to families, but of providing parents and adolescents with information and support on how best to strengthen their relationships, manage their emotions and maintain open communication channels in order to assist the young people with T1D in their transition to the independent self-management of their condition.

## Competing interests

The authors have no competing financial or non-financial interests.

## Authors’ contributions

SMM wrote the paper, did the data analysis and was involved in all stages of the project’s conception, design and implementation. NJH was the Chief Investigator for the project. She reviewed this paper and took the major role in all stages of the project’s conception, methodological design, data collection, analysis and interpretation. VEH was involved in the interpretation of data analyses and writing of the paper. EPN was involved in the project’s conception and design, and gave feedback regarding data analysis and writing. FJC was involved in the project’s conception, design and implementation, especially through the diabetes clinics. He gave specialized advice on the medical aspects of the project and this paper. All authors have read and approved the final manuscript.

## Authors’ information

SMM is Emeritus Professor in Psychology at Swinburne University. Her research interests include psychology of developmental transitions, health psychology and risk-taking behaviour. NJH is a Senior Research Fellow at the Parenting Research Centre and Adjunct Research Fellow at Swinburne University. Her research interests include health psychology, child development and online delivery of psychological services. VEH is a Research Fellow at the Parenting Research Centre. Her research interests are in health psychology, and parenting confidence and wellbeing. EPN holds appointments in psychology at the Royal Children’s Hospital and the University of Melbourne and is a Senior Research Fellow in the Murdoch Children’s Research Institute. She has extensive experience in clinical research, focusing on the neuro-cognitive and psychosocial impact of type 1 diabetes in children. FJC has been the Head of Diabetes Services and Deputy Director of the Department of Endocrinology and Diabetes at The Royal Children’s Hospital Melbourne and Professorial Fellow within the Department of Paediatrics, University of Melbourne.
